# Consideration of Yarn Anisotropy in the Investigation of the Puncture Resistance of Fibrous Materials

**DOI:** 10.3390/polym14050883

**Published:** 2022-02-23

**Authors:** Chao Luo, Ye Sun, Kaoru Wakatsuki, Hideaki Morikawa, Limin Bao

**Affiliations:** Faculty of Textile Science and Technology, Shinshu University, Nagano 3860018, Japan; 20hs112g@shinshu-u.ac.jp (C.L.); 20hs107a@shinshu-u.ac.jp (Y.S.); kaoruw@shinshu-u.ac.jp (K.W.); morikaw@shinshu-u.ac.jp (H.M.)

**Keywords:** anisotropy, radial-direction stiffness, finite element analysis, puncture resistance

## Abstract

High-performance yarns are widely used to produce protective fabrics, including stab-resistant materials. The most common approach to studying the mechanism of puncture prevention is to use simulation to assist analysis. However, the anisotropy of the yarn is often overlooked during simulation owing to various factors. In fact, there is a marked difference between the axial and radial properties of a yarn. This may lead to large errors in research. In the present study, a composite material with a grid structure for puncture analysis was designed to investigate the influence of yarn anisotropy on the accuracy of simulation results. The present study combined an actual experiment with a simulation. In the actual experiment, Kevlar yarn/epoxy resin was used to prepare a mesh composite with a spacing of 1 mm. In the simulation, a 1:1 simulation model of composite material was established using finite element software. A simulated puncture experiment was conducted based on the actual experimental conditions and material parameters. After considering yarn anisotropy, the simulation results were closer to the actual experimental results. The simulation revealed that the main failure modes of the mesh material were the fracture of the resin and the bending deformation of the yarns at the junctions, while the surrounding areas were almost unaffected.

## 1. Introduction

With the globalization of the war on terror and increased awareness about the need for protection, personal safety has become an important issue. The use of firearms is severely restricted in many countries, but the threat from sharp objects used as weapons [1] is ubiquitous. Police and security guards in particular often encounter violent situations. Most existing anti-stab armor [2] is made from steel plates, which are very heavy. This greatly inconveniences the wearer and inhibits their ability to perform their tasks. Therefore, increasing attention has been paid to the development of new puncture-resistant materials.

In the field of lightweight protective materials, aramid fibers are essential. Due to their excellent performance, they have made great contributions in many fields. Zheng et al. [3] used the principle of bionics to develop and prepare three-dimensional Kevlar polyimide composite materials. Excellent three-dimensional structure makes it a composite with ultra-light, superior anti-compressive and flame-retardant properties. Zhou et al. [4] proposed to develop a high-performance multi-purpose composite material. Materials prepared using Kevlar fibers, shear-hardening gels, and MXene exhibit excellent thermal management and intelligent safeguarding.

Based on Kevlar fibers, many experts have carried out research on protective materials. Usman Javaid et al. [5] studied the effect of changes in the surface friction of the fabric and the knife penetration angle on quasi-static knife penetration resistance. Zhang et al. [6] put forward the relationship between the slippage of Intralayer interface and the anti-impact performance. It was found that the impact velocity and constraint conditions were the two main influencing factors. Sy et al. [7] used flax fiber and Kevlar fiber as raw materials to prepare epoxy resin matrix composite materials. The impact resistance of materials under different layup methods has been studied. In order to improve the impact resistance of Kevlar composites, Eltaher et al. [8] prepared CFRP/Kevlar sandwich composites. The results show that the residual compressive properties of the sandwich composites are greatly improved. White et al. [9] considered that all armor depends on a combination of weight, flexibility, and protection. Guleria et al. [10] prepared Kevlar composites by improving the molding method of the material, that is, using microwave-assisted molding. The mechanical properties of the materials prepared by this method are improved. In terms of impact, different impact head shapes also have a big impact on performance. Karamooz et al. [11] found that among the hemispherical, conical, and flat shapes, the highest energy absorption and damage area were obtained with the conical impactor. As 3D printing technology matures, researchers also apply it to the field of stab prevention. Hetrick et al. [12] proposed the technology of continuous Kevlar fiber reinforced 3D printing composites. This material was used to study the effects of different fiber modes, stacking modes, and fiber orientations on the impact properties of the material.

It is difficult to observe and analyze the material failure mechanism in the puncture experiment. Therefore, it is necessary to carry out auxiliary analysis with finite element analysis software. Hou et al. [13] established two different finite element models to describe the tensile properties of nonwovens, and macroscopically assessed the influence of thermal bonding points on the deformation mechanism and behavior of the materials. Ridruejo et al. [14] used a combination of experiment and simulation to determine the microscopic mechanism of deformation and damage in a nonwoven glass fiber mat. Zeng et al. [15] used the finite element method to study the effects of yarn inclination, yarn friction, and tensile modulus on yarn properties. Zhou et al. [16] used the finite element method to study the blasting performance of fabric-reinforced rubber composites and found that the rubber improved the breaking strength of the fabric. Sun et al. [17] tested the puncture behavior of various structures using finite element analysis. Puncture damage involves three stages: tensioning of the fabric, slippage of the weft/warp yarn, and breakage of the yarn. Zhu et al. [18] used finite element analysis to study the stab prevention capabilities of fish scales and found that the structure and behavior of natural fish scales offer effective protection against several types of threat. Lian et al. [19] simulated the dynamic performance of three-dimensional braided composites and found that they offered protection against mild impact. Termonia [20] used models to study the exact mechanism of fabric puncture, which comprise four stages: contact, penetration, friction, and slip.

At the same time, we have also carried out related experiments and have accumulated some experience. Bao et al. [21] developed a new high-density nonwoven structure to enhance the stab resistance of protective clothing. They found that material prepared by single-layer hot pressing guarantees puncture resistance and improves comfort. Bao et al. [22] tested the stab resistance of aramid fabrics comprising nanoparticles. They found that the puncture performance of the material increased as the particle diameter decreased, and the friction of the intersecting parts of the plain weave fabric had the greatest impact on puncture resistance. Chuang et al. [23] studied the recycling of fiber materials and used nonwoven technology to prepare mixed fiber fiberboards. They reported that the material had higher puncture performance than traditional woven fabrics.

As can be seen from the research described above, many different structures and materials are used to make protective armor. Among these materials, high-performance fiber composites are currently the focus of intense research because they are lightweight and offer good protection. Puncture is a transient process, so it is difficult to study the mechanism by which it occurs in fabrics through practical experiments alone. To explore in depth the mechanism by which punctures occur, it is usually necessary to carry out auxiliary simulation experiments. However, during simulation experiments it is easy to overlook the anisotropy of the inner yarn of the composite material, which is usually taken to be an isotropic material for finite element analysis. As is well known, yarn is an anisotropic material, and there is a big difference between its axial performance and its radial performance. If the yarn is regarded as an isotropic material, it may cause a large error in the calculation and analysis.

In the present puncture analysis, grid structure materials were specially designed to take into account the influence of anisotropy on the simulation results. The yarns were arranged in parallel, which avoided the uncertainty associated with the interlaced yarns of common fabrics, such as those comprising plain or twill structures. This was more conducive to the analysis of the experimental results. The results will provide a theoretical reference for the preparation of stab-resistant materials and will lay a solid foundation for further research.

## 2. Puncture Experiment

### 2.1. Experiment Preparation

**Figure 1:**
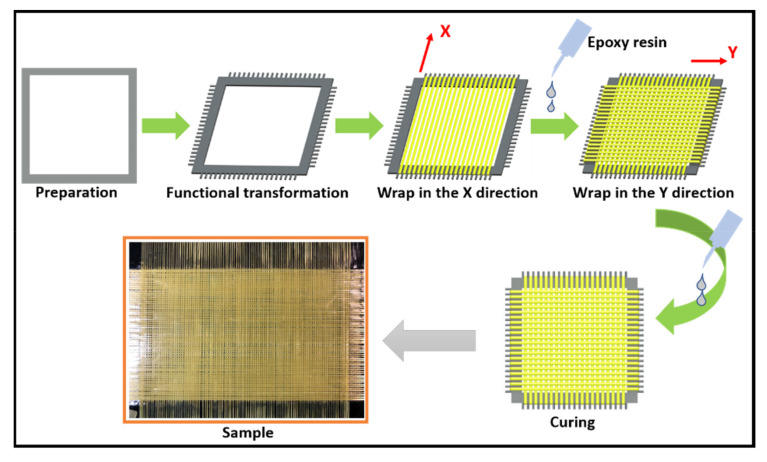
Preparation of the composite sample.

The grid structure material was designed for puncture analysis. The sample preparation process is shown in the Figure 1. First, the metal frame was pretreated to remove impurities and dust on its surface. The treated frame was then modified and zigzagged fixed grooves were installed around it. After transformation, the spacing of the metal frame sawtooth was 1 mm. The X-direction yarn was then wrapped and coated in epoxy resin, which was cured. Finally, the yarn was wound in the Y direction and coated in epoxy resin, which was also cured. The yarns were made of Kevlar (Teijin Ltd., Tokyo, Japan), and the epoxy resin was obtained from the Nagase ChemteX Corporation (Tokyo, Japan).

**Figure 2:**
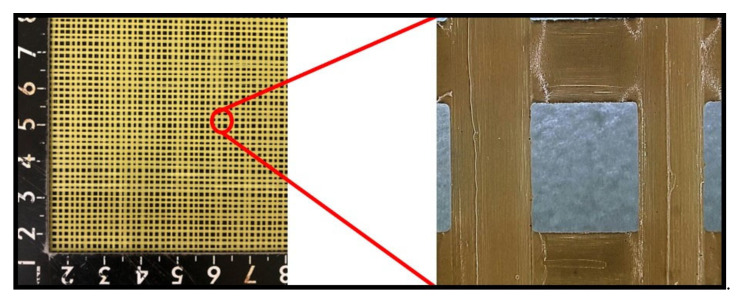
Photographs of the composite sample and the partial amplification.

Figure 2 illustrates the partial amplification and the prepared sample. As shown, the resin was evenly distributed on the yarn and the X-direction yarns combined well with the Y-direction yarns.

### 2.2. Determination of Material Parameters

After sample preparation, the parameters of the material were determined. Because the yarn in the material had been compounded with the resin to form a composite material, when we were ready to test the yarn parameters, the yarn composite material was prepared by the same method, which included determination of the axial and radial properties of the material.

#### 2.2.1. Measurement of Axial Modulus

**Figure 3:**
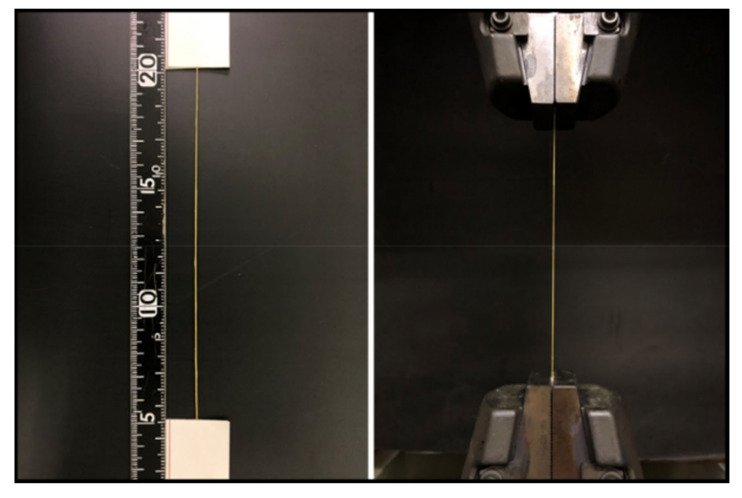
Determining the tensile mechanical properties of the Kevlar yarns.

Please refer to test standard ASTM D7269 [24] for a description of the determination of the axial modulus of the yarn. The prepared yarn resin composite material is shown in the Figure 3. Spacers were installed on both ends of the yarn to prevent the material from slipping and to avoid stress concentration during the pulling process.

**Figure 4:**
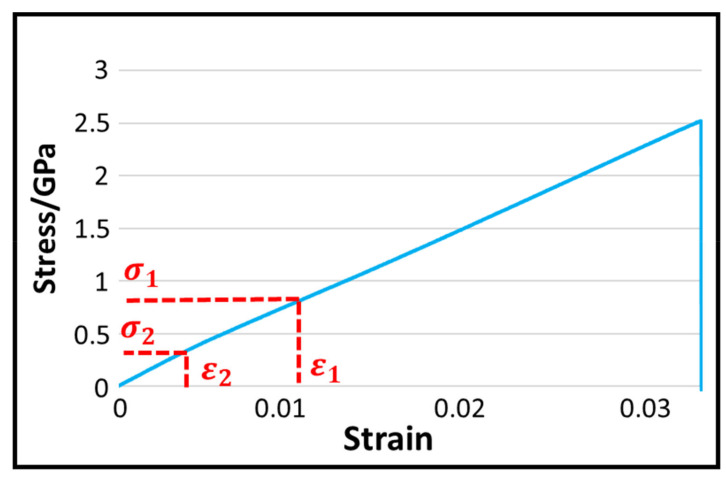
Tensile mechanical properties of the Kevlar yarns.

According to Formulae (1)–(4), the yarn stress-strain curve was drawn, and the elastic modulus of the material was calculated. The tensile mechanical properties of the Kevlar yarns are shown in Figure 4. (*σ*: tensile stress, *F*: tensile strength, *A*: cross-sectional area of yarn, *T*: linear density of yarn, 1: yarn density, *ε*: tensile strain, *L*: length of yarn after stretching, *L*_0_: yarn length, *E*: tensile elastic modulus)

(1) $$\sigma =\frac{F}{A}$$

(2) $$A=\frac{T}{D}$$

(3) $$\varepsilon =\frac{L-L_{0}}{L_{0}}$$

(4) $$E=\frac{\sigma_{1}-\sigma_{2}}{\varepsilon_{1}-\varepsilon_{2}}$$

#### 2.2.2. Measurement of Radial Modulus

**Figure 5:**
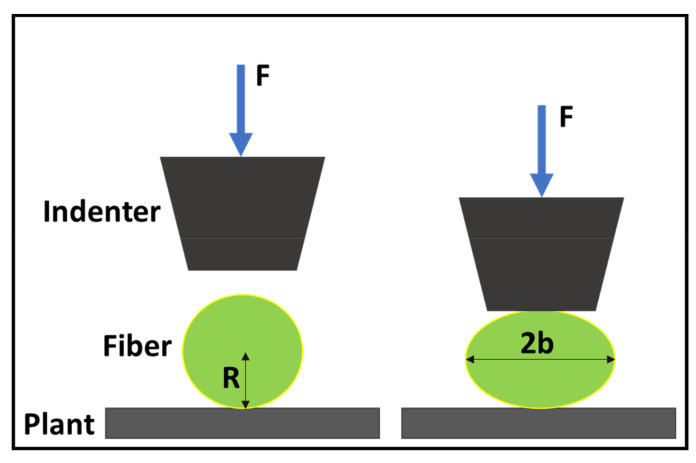
Schematic diagram of the transverse tester.

Because there is no special test standard for the radial modulus of a yarn, we referred to the test method used for the radial performance of a single fiber. As shown in Formulae (5) and (6), the radial modulus test refers to the method described by Professor Kawabata [25], which can be seen in Figure 5.

(5) $$U={\left(4F∕\pi \right)}^{\;}\left[\left(1∕E_{T}\right)-\left(\nu_{LT}^{2}∕E_{T}\right)\right]\left[0.19+\sin h^{-1}\left(R,b\right)\right]$$

(6) $$b^{2}=\left(4FR∕\pi \right)\left[\left(1∕E_{T}\right)-\left(\nu_{LT}^{2}∕E_{T}\right)\right]$$

where:

*U*: the change in fiber diameter;
*F*: the compressive force;
*E_T_*: the transverse modulus of the fiber;
ν_*LT*_: the longitudinal Poisson’s ratio of the fiber;
*R*: the radius of the fiber; and
*b*: the contact width of the fiber during compression.

**Figure 6:**
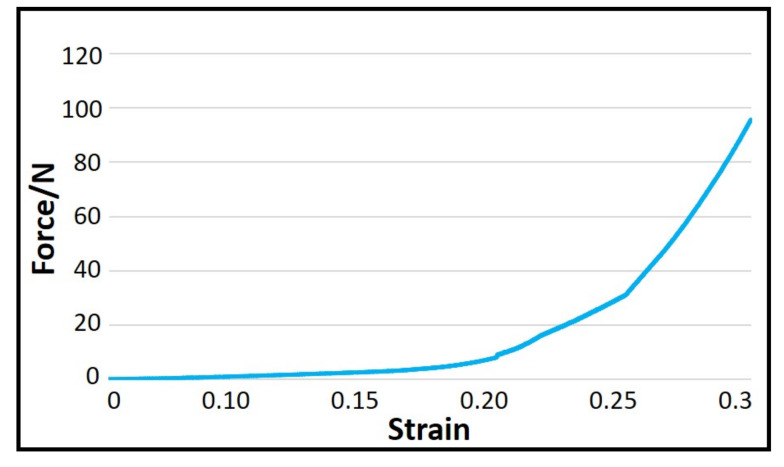
Transverse compressive curve of the Kevlar yarns.

**Figure 7:**
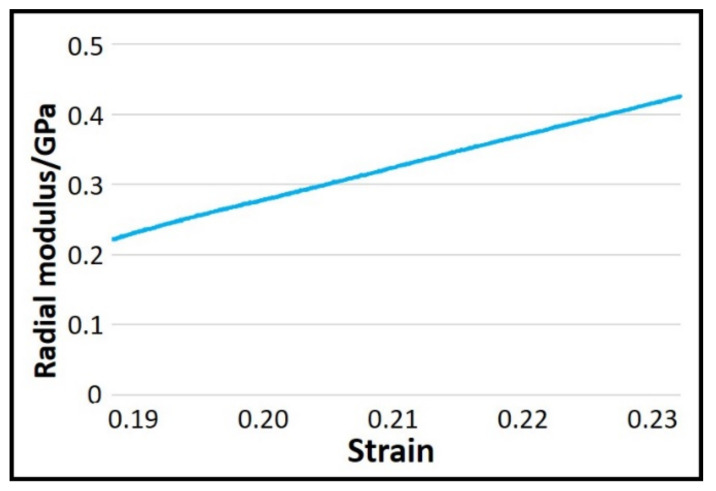
Radial modulus of the Kevlar yarns.

Owing to the limitations of the laboratory equipment, it was impossible to accurately measure all the parameters required in the formulae in the actual test. Therefore, using the results from previous research [26], the formulae described above were further simplified to Formula (7). Yarn compression experiments were carried out according to the parameter requirements of Formula (7). The experimental results are shown in Figure 6, and the radial modulus was obtained based on this result (Figure 7).

(7) $$E_{T}=F\pi ∕16RL^{2}\varepsilon^{2}$$

where:

*E_T_*: the transverse modulus of the fiber;
*F*: the compressive force;
*R*: the radius of the fiber;
*L*: the length of the fiber during compression; and
ε: the strain of the fiber.

According to the above-mentioned test methods for the elastic modulus of the material in the axial and radial directions, the elastic modulus of the material was calculated as shown in Table 1 by using Equations (1) and (2),

### 2.3. Puncture Experiments

**Figure 8:**
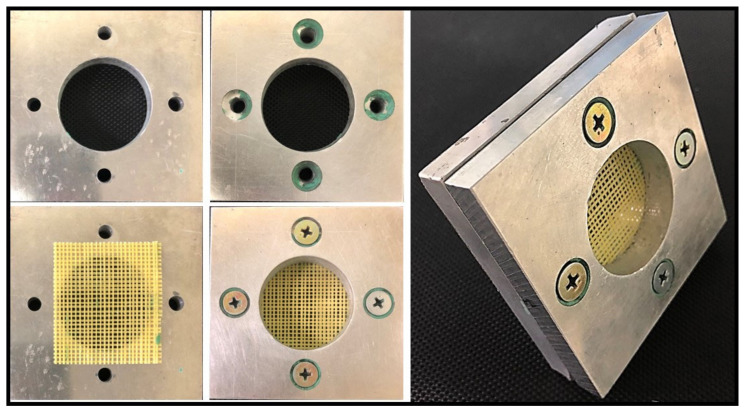
Preparation of the puncture sample.

The prepared sample was fixed on the puncture mold, as shown in Figure 8. The mold was divided into upper and lower pieces, both fixed by screws to prevent slipping.

**Figure 9:**
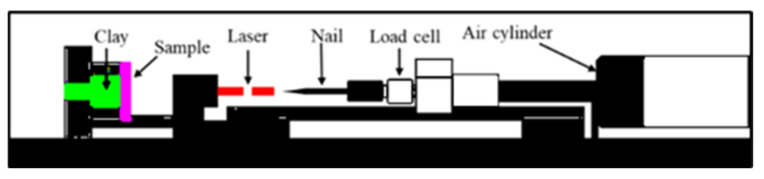
Puncture test machine.

A schematic diagram illustrating the puncture experiment [27] is shown in Figure 9. The sample was fixed at the left clay, and the puncture needle was fixed on the air jet device. The puncture speed was controlled by adjusting the air valve. The tail of the needle was connected to a pressure sensor to determine the puncture force. A laser lamp was used to determine the displacement of the needle.

The puncture speed was 2 m/s in this experiment. Five samples were prepared to ensure the accuracy of the result.

### 2.4. Dynamic Analysis of the Puncture Results

**Figure 10:**
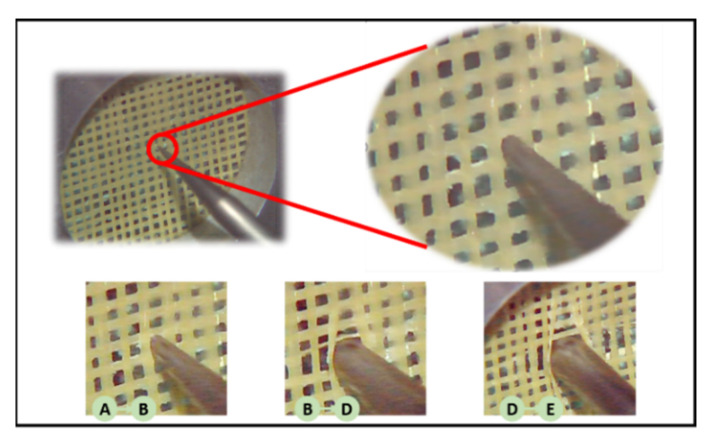
The composite puncture experiment. (The three pictures AB, BD, and DE in the figure correspond to the three stages in Figure 11).

**Figure 11:**
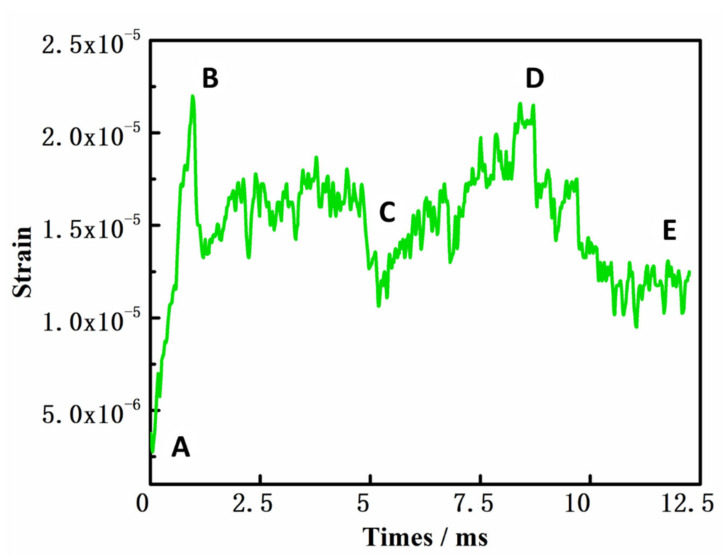
Results of the composite puncture experiment (The curves marked A to E in the figure represent different stages in the puncture process.).

As shown in Figure 10, the grid material puncture experiment was divided into five parts (A–E) and the corresponding strain curve is shown in Figure 11. In the first stage (part A)—before the puncture needle had touched the material—the curve was relatively flat; the needle had not yet been subjected to force and the strain was constant. In the second stage (part B), the needle came into contact with the grid structure, the extrusion force on the yarn gradually increased, and the strain began to rise as the needle penetrated deeper. At this time, the yarn began to exhibit slight bending deformation and the square holes gradually expanded into circular holes. The strain reached the first peak and then dropped rapidly when the yarn peeled off at the weakest point of the four crossing points. In the third stage (part C), the needle continued to penetrate the material after the yarn had separated at the intersection of the first ring. As the diameter of the needle continued to expand, the circle of fibers around the needle was subjected to friction and extrusion, and the curve therefore continued in a straight line until the four intersection points of the yarn fell off. The extrusion force on the yarn decreased and the strain also decreased again. In the fourth stage (part D), the needle continued to move forward after it had broken through the first ring. When the diameter of the needle increased, the yarn continued to be squeezed to both sides. At the same time, the cross points between the yarns prevented their expansion again. Therefore, the strain increased again until it reached its maximum. The yarn at the cross point peeled off, the bending degree of the yarn reached the diameter of the needle, the depth of the needle no longer increased the expansion force of the yarn, and the force between the needle and the yarn reached dynamic equilibrium, so the strain decreased and remained constant.

The analysis provided above indicates that when the needle was inserted, the yarn was mainly subjected to the extrusion and friction forces at the contact point with the needle. The yarn at the intersection was peeled off revealing that the material had been punctured.

## 3. Simulation Experiment

### 3.1. Model Establishment

With regard to the actual experimental materials, the 1:1 structure model—including the composite grid materials, the upper/lower splints, and the piercing needles—was established using SolidWorks software (SolidWorks Premium 2016, Dassault Systèmes SolidWorks Corporation, Concord, MA, USA). To simplify the simulation calculation, the following assumptions were made about the composition and structure of the composite grid materials:

(1) the yarns were continuous filaments with uniform bars, and the cross section was a runway type; and
(2) the composite material had no defects and the resin distribution was uniform [28].

**Figure 12:**
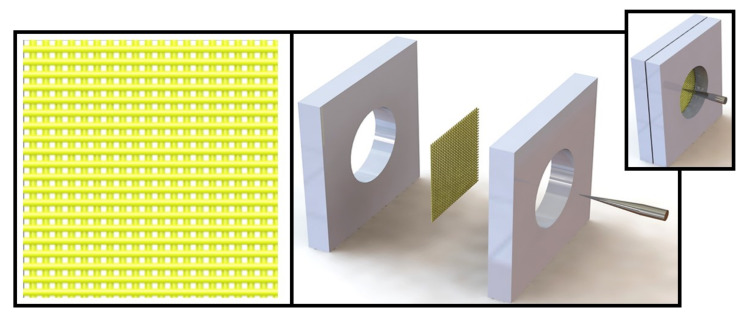
Illustration of geometrical puncture simulation model.

The model explosion diagram and assembly diagram (upper right) shown in Figure 12 are based on these assumptions.

### 3.2. Model Parameters

The simulation software used in this experiment is ANSYS. Using the part of explicit dynamics [29], a finite element simulation of the puncture experiment was performed. The model was imported into the finite element software after being established by SolidWorks. There is no Kevlar yarn parameter in the engineering parameter database in the software, so it was necessary to customize the material parameters. We created a new material parameter library and selected the Anisotropic Material option. The warp and weft parameters of the yarn were set according to the values in Table 1. Therefore, the anisotropy of the yarn plays an important role in the numerical simulation.

The model was then meshed. To reduce computation time, the meshes involved in the piercing contact were closely divided, whereas the meshes around the model were relatively sparse [30]. The mesh size of the central area was 0.01 mm, and the surrounding part was 0.05 mm by adopting the type of Hex Dominant. After division, there were 288,301 nodes and 112,468 elements.

The model was fixed by referring to the material-fixing method in the actual experiment. The upper and lower clips were set to fixed, and the contact between the yarns and the upper and lower clips were set to bonded. The interfacial contact between the yarns was also set to bonded. At the same time, in order to simulate the failure during puncture, the maximum strain failure criterion was set for the yarns part. The puncture speed was set to 2 m/s, and the contact between the blade and the sample was introduced as a general contact with a friction coefficient of 0.2 [31].

For comparative experiments, the parameters were imported without considering material anisotropy and the simulation experiments were carried out under the same conditions. The radial and axial parameters of the yarn in this model were the same and were used to assess how much yarn anisotropy affects the simulation results.

The simulated puncture experiment was carried out after all the settings had been completed.

### 3.3. Puncture Simulation

#### 3.3.1. Comparison of Simulation Results

**Figure 13:**
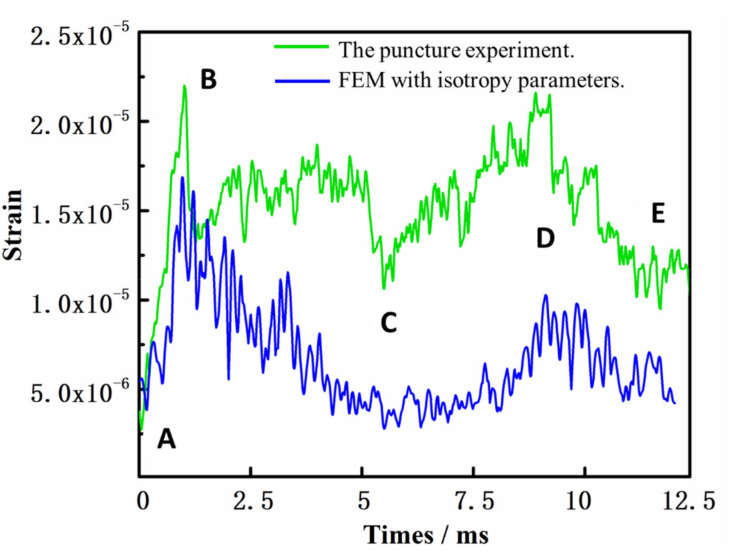
Strain results obtained using the finite element method (FEM) with isotropy parameters. (The curves marked A to E in the figure represent different stages in the puncture process.).

**Figure 14:**
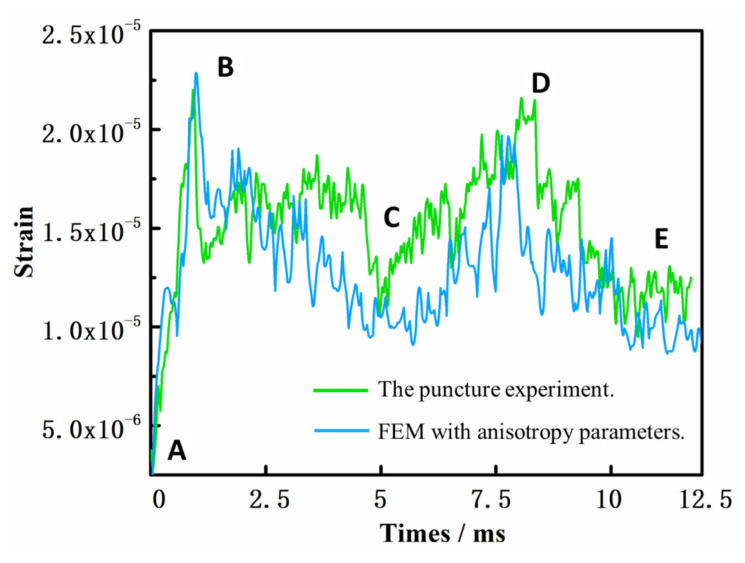
Strain results obtained using the finite element method (FEM) with anisotropy parameters. (The curves marked A to E in the figure represent different stages in the puncture process.).

**Figure 15:**
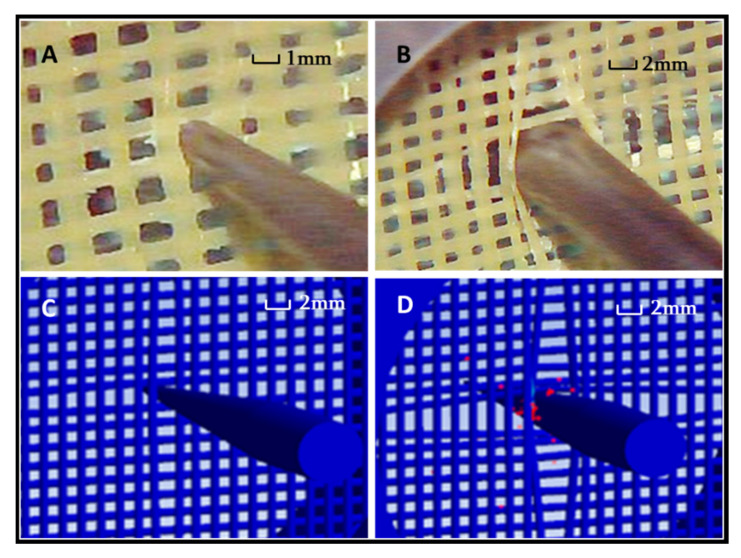
Comparisons between the results obtained using the finite element method (FEM) with anisotropy parameters and the experimental results. ((**A**) shows the needle contacted material in the experiment, (**B**) shows the needle completely penetrated the material in the experiment, (**C**) shows the needle contacted material in the simulation experiment, (**D**) shows the needle completely penetrated the material in the simulation experiment).

The results of the puncture simulation experiment are shown in Figure 13 and Figure 14. The failure morphology between the experiment and simulation is shown in Figure 15. As shown in Figure 13, the simulation results obtained with the isotropic parameter settings were quite different from the actual results. Although the overall trend of the curve was similar to the actual experimental results, the maximum value was quite different. The strain was generally small between points C and D. This is because when the yarn is regarded as an isotropic material, its radial characteristics are stronger. Therefore, the numerical values shown in the strain curve were quite different from the experimental values.

However, compared with the differences between the curves in Figure 11 and Figure 14, the simulation results using yarn anisotropy parameters were more accurate. The strain curve of the simulation experiment also included five stages, the trend was the same as that in the actual experiment, and the value was within the allowable error range. The difference between the simulation and experimental results occurred in stage B. This was probably due to manual errors in the preparation of the experimental materials. The connection strength of the four crossing points around the grid was not the same, so there was a certain time difference between stripping and shedding. The maximum strain during stage B decreased and the strain subsequently adopted a straight trend for a period of time. The connection strength of the four crossing points was the same in the simulation experiment, so the strain decreased directly and the fluctuation was more obvious after the maximum strain of the B stage was reached. At the same time, the failure morphology of the simulated experiment under this condition is also consistent with the failure morphology of the actual experiment in the Figure 15.

In summary, the simulation results were closer to the actual experiment results after considering yarn anisotropy. Therefore, the simulation results had a certain reference value, so a detailed analysis of the material was carried out.

#### 3.3.2. Microscopic Analysis of the Simulated Puncture Results

(1) Stress analysis of the whole fabric

**Figure 16:**
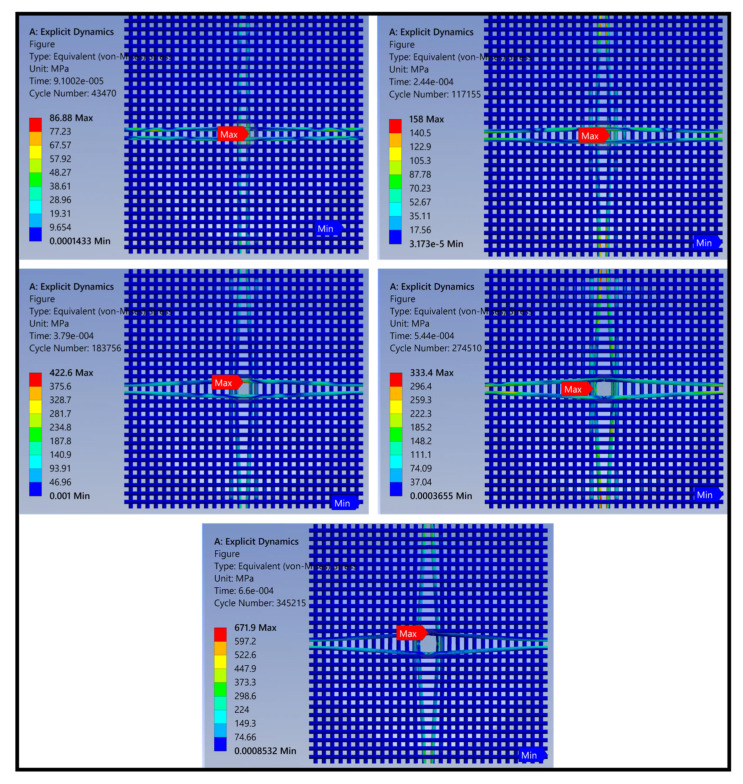
Damage morphologies obtained from the finite element method (FEM) results (stress diagram of the overall material).

The upper and lower collets and puncture needles in the model were hidden, and the intermediate material was extracted for analysis. The overall stress nephogram of the grid composite obtained from the puncture load simulation results is shown in Figure 16. Different colors in the figure show different stress levels of the material.

The figure reveals that when subjected to puncture load, the yarn stress around the mesh at the puncture site began to increase. As the penetration depth of the needle increased, the yarn began to deform and bend and the stress wave spread rapidly along the transverse and longitudinal directions of the yarn. Under the action of the stress wave, the material absorbed energy through deformation bending, resin fragmentation, and fiber fracture. Until the needle penetrated the material, the yarn deformation bending reached the maximum and the stress value was not increasing.

During the whole process of puncture simulation, the stress values of the yarn were largest in the longitudinal and transverse directions. This was the main influencing factor of the puncture effect [32] and the yarn around the material was largely unaffected. Therefore, the yarns in the longitudinal and transverse directions were extracted for further refinement analysis.

(2) Stress analysis of central yarns

**Figure 17:**
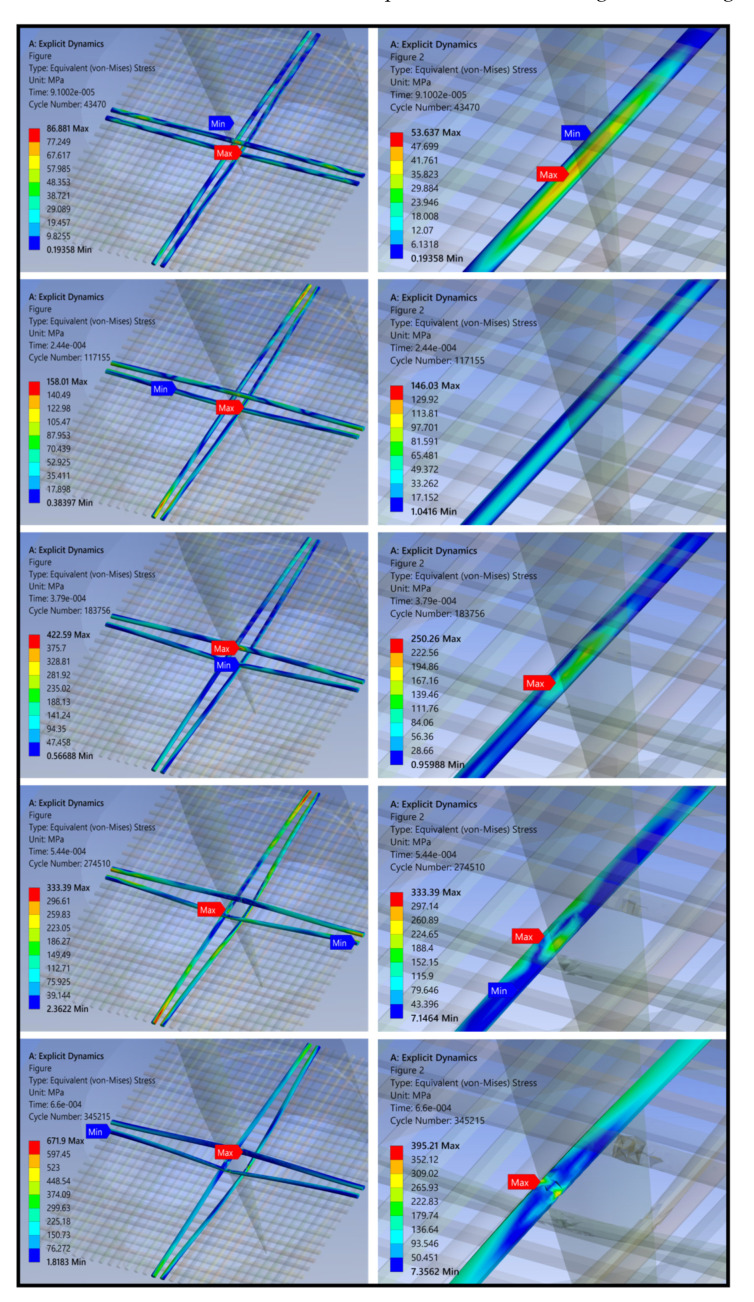
Damage morphologies obtained from the finite element method (FEM) results (stress diagram of yarn parts).

The yarn stress nephogram at the puncture center is shown in the Figure 17. The stress value of the outer yarn in contact with the needle was greatest when the material was subjected to puncture injury, whereas the stress value of the inner yarn was smaller. As the penetration depth of the needle increased, the stress value of the inner yarn gradually increased. At that time, the yarn began to bend slightly and the maximum value occurred following contact with the needle. The puncture depth increased further and the stress value also increased further. At that time, the maximum stress point was located at the intersection of the two yarns, and the bending degree of the yarns increased further. When the needle was completely inserted, the degree of yarn bending reached a maximum. At that time, the maximum stress point was located at the contact point with the needle and the material at the contact point had a certain degree of damage.

The analysis described above suggests that when subjected to puncture, the yarn at the contact point with the needle and the adjacent yarn intersection were the parts with the largest stress values, and the diffusion stress values along both sides of the yarn axis gradually decreased. The surrounding parts were largely unaffected by the puncture. Yarn deformation and resin peeling were the main modes of damage. When the resin at the intersection peeled off, yarn bending deformation became easier, the needle penetrated completely, and the material was ultimately destroyed.

## 4. Conclusions

In the present study, details that are easily overlooked in a simulation experiment were explored. In simulation experiments, the anisotropy of the yarn is often neglected owing to various factors. There is a marked difference between the axial and radial properties of a yarn. This may lead to serious research errors. A grid structure composite material was designed to study the influence of yarn anisotropy on the accuracy of puncture simulation. The results revealed that a simulation that took yarn anisotropy into account was closer to the actual experimental results than a simulation that did not. Numerical simulation can reveal the stress change inside a material during puncture, and the specific puncture mechanism can be displayed visually. The experimental results demonstrated that the main failure modes of the mesh material were resin fracture at the junction, bending deformation of the yarn, and slippage between the fiber and the resin.

Based on this experimental model, the factors affecting the puncture performance of materials will be investigated further, and the results will inform future research into fabrics and nonwoven structures.

## Figures and Tables

**Table 1:** Radial and axial parameters of the Kevlar yarns.

	Young’s Modulus (GPa)	Tensile/Compression Strength (GPa)	Failure Strain
Axial direction of yarn	78.62 ± 2.54	2.58 ± 0.32	0.032 ± 0.0024
Radial direction of yarn	0.32 ± 0.04	0.23 ± 0.02	0.263 ± 0.031
